# Trends in Management of Children With Acute Gastroenteritis in US Emergency Departments

**DOI:** 10.1001/jamanetworkopen.2022.11201

**Published:** 2022-05-10

**Authors:** Brett Burstein, Sarah Rogers, Terry P. Klassen, Stephen B. Freedman

**Affiliations:** 1 Division of Pediatric Emergency Medicine, Department of Pediatrics, Montreal Children’s Hospital, McGill University Health Centre, Montreal, Quebec, Canada; 2 Department of Epidemiology, Biostatistics and Occupational Health, McGill University, Montreal, Quebec, Canada; 3 Department of Pediatrics, Health Sciences Center, Children’s Hospital Winnipeg, University of Manitoba, Winnipeg, Manitoba, Canada; 4 Children’s Hospital Research Institute of Manitoba, University of Manitoba, Winnipeg, Manitoba, Canada; 5 Sections of Pediatric Emergency Medicine and Gastroenterology, Departments of Pediatrics and Emergency Medicine, Alberta Children’s Hospital Research Institute, Cumming School of Medicine, University of Calgary, Calgary, Alberta, Canada

## Abstract

This repeated cross-sectional analysis examines national trends in emergency department (ED) treatment of children with acute gastroenteritis.

## Introduction

Acute gastroenteritis (AGE) is a common reason for emergency department (ED) visits and hospitalizations for children.^1^ Recommendations emphasize oral rehydration therapy (ORT) for children with AGE and mild to moderate dehydration, and intravenous rehydration for those with severe dehydration or in whom ORT fails.^2^ Intravenous rehydration is associated with longer hospital stays, return visits, adverse events, costs, and pain. Oral ondansetron reduces vomiting, the need for intravenous rehydration, and hospitalizations; however, benefits are limited to children with evidence of dehydration.^3^ Reducing unnecessary interventions and admissions is crucial to optimizing outcomes. Given the high prevalence and costs associated with AGE treatment for children, understanding national trends in management in a broad, generalizable sample is important.

## Methods

This repeated cross-sectional analysis used data from the National Hospital Ambulatory Medical Care Survey (NHAMCS) ED database from January 1, 2006, to December 31, 2018, undertaken following release (June 2021). The NHAMCS is conducted annually using multistage probability sampling of approximately 30 000 ED visits in the US to generate nationally representative, population-level estimates.^4^ We analyzed visits for patients younger than 18 years with had a primary discharge diagnosis of AGE or a primary diagnosis of nausea, vomiting, diarrhea, or dehydration and a secondary diagnosis of AGE (eTable in the Supplement), as described previously.^5^ Discharge diagnoses were identified using *ICD-9-CM* (2006-2015) and *ICD-10-CM* (2016-2018) codes. Patient- and ED-level variables were extracted, including diagnostic testing and treatments. Survey-weighting procedures were applied to estimate annual proportions. Trends were analyzed using a weighted Pearson χ^2^ test of proportions (Stata, version 14.1; StataCorp LLC). A 2-tailed *P* < .05 was considered significant. This study was exempted from review by the McGill University Health Centre Research Ethics Board because the NHAMCS data are deidentified and publicly available. This study followed the Strengthening the Reporting of Observational Studies in Epidemiology (STROBE) reporting guideline.

## Results

The NHAMCS contained 81 757 unweighted ED visits for pediatric patients between 2006 and 2018; 4122 patients (mean age, 4.8 years; 95% CI, 4.6-5.1 years) met inclusion criteria, representing an estimated 19.87 million (95% CI, 17.74 to 22.01 million) visits for children with AGE (Table). Most visits were to nonacademic (84.9%; 95% CI, 81.4%-87.8%) and nonpediatric (80.4%; 95% CI, 74.7%-85.1%) EDs. Visits for AGE increased over time (2006: 1.23 million; 95% CI, 1.01-1.46; 2018: 1.87 million; 95% CI, 1.29-2.45; *P* = .03 for trend) (Figure) and as a proportion of all ED visits by children (2006: 4.7%; 95% CI, 4.1%-5.4%; 2018: 5.6%; 95% CI, 4.6%-6.7%; *P* = .02 for trend). Ondansetron administration increased from 10.6% (95% CI, 7.0%-15.7%) to 59.2% (95% CI, 52.0%-66.1%; *P* < .001 for trend). Intravenous rehydration and hospitalizations were both unchanged over time (28.2% and 4.8% in 2018, respectively). Ondansetron was administered to 53.9% (95% CI, 49.4%-58.3%) of children who received intravenous fluids and 49.1% (95% CI, 35.7%-62.6%) of those hospitalized.

**Table.** Characteristics and Outcomes Among Children With AGE Presenting to US EDs, 2006-2018

Characteristic or outcome	Unweighted observations, No.	Population estimate, in millions (95% CI)	Overall weighted visits, % (95% CI)
Visits			
Total ED visits by children with AGE	4122	19.87 (17.74-22.00)	5.1 (4.9-5.4)^a^
AGE with nausea and vomiting	2774	14.0 (12.4-15.6)	70.6 (68.4-72.7)
Age group, y^b^			
0-<3	1977	9.32 (8.20-10.43)	46.9 (44.3-49.5)
3-<13	1663	8.14 (7.17-9.11)	41.0 (38.8-43.1)
13-<18	482	2.41 (2.01-2.82)	12.1 (10.7-13.8)
Sex			
Female	2011	9.81 (8.59-11.02)	49.4 (46.9-51.8)
Male	2111	10.07 (8.94-11.19)	50.7 (48.2-53.1)
Race			
Black	1065	5.17 (4.26-6.07)	26.0 (23.1-29.2)
White	2758	13.62 (12.13-15.11)	68.6 (65.3-71.6)
Other^c^	299	1.08 (0.84-1.33)	5.5 (4.4-6.7)
Insurance provider			
Medicaid or Medicare	2448	11.80 (10.18-13.42)	59.4 (56.1-62.6)
Private	1095	4.99 (4.39-5.58)	25.1 (22.9-27.5)
Self-pay	225	1.13 (0.89-1.37)	5.7 (4.6-7.0)
Other or unknown	354	1.96 (1.46-2.45)	9.9 (7.8-12.5)
Triage acuity level			
Immediate or emergency	208	0.83 (0.64-1.02)	4.2 (3.4-5.2)
Urgent	1695	8.04 (7.09-8.99)	40.5 (37.6-43.4)
Semiurgent	1234	6.09 (5.26-6.92)	30.7 (28.0-33.5)
Nonurgent	239	0.99 (0.71-1.27)	5.0 (3.8-6.4)
Unknown or unavailable	746	3.93 (3.06-4.79)	19.8 (16.5-23.4)
Type of institution^d^			
Pediatric	787	3.89 (2.68-5.10)	19.6 (14.9-25.3)
Nonpediatric	3335	15.98 (14.23-17.73)	80.4 (74.7-85.1)
Teaching	826	3.00 (2.30-3.71)	15.1 (12.2-18.6)
Nonteaching	3296	16.87 (14.92-18.82)	84.9 (81.4-87.8)
Diagnostic testing			
Any blood investigations	1308	6.15 (5.39-6.91)	30.9 (28.5-33.5)
Any diagnostic imaging	669	3.63 (3.05-4.22)	18.3 (16.4-20.3)
Ondansetron use			
ED administration	1581	8.85 (7.59-10.11)	44.6 (41.5-47.7)
Intravenous rehydration			
Overall	1077	5.19 (4.56-5.83)	26.1 (24.1-28.3)
Received ED ondansetron	516	2.80 (2.35-3.24)	53.9 (49.4-58.3)
Hospitalization			
Overall	146	0.65 (0.46-0.84)	3.3 (2.5-4.3)
Received ED ondansetron	55	0.32 (0.18-0. 46)	49.1 (35.7-62.6)

Abbreviations: AGE, acute gastroenteritis; ED, emergency department.

^a^ Percentage of all visits for patients younger than 18 years in the National Hospital Ambulatory Medical Care Survey (NHAMCS).

^b^ Mean age of 4.8 years (95% CI, 4.6-5.1 years).

^c^ The race variable is captured by NHAMCS site representatives as 1 of the following: White, Black/African American, Asian, Native Hawaiian/other Pacific Islander, American Indian/Alaska Native, or more than 1 race reported. The NHAMCS then recategorizes race as White, Black, or other. The recategorized race variable was used for the analyses of this study.

^d^ Emergency departments were classified as pediatric hospitals if 85% or more of all visits were for patients younger than 21 years and classified as teaching hospitals if 25% or more of all patients were evaluated by a resident physician.

**Figure.**
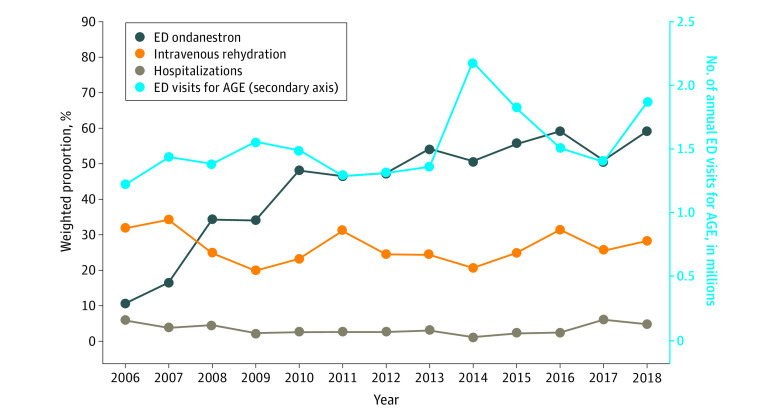
Annual Trends in US Emergency Department (ED) Visits, Ondansetron Use, Intravenous Rehydration, and Hospitalizations for Children With Acute Gastroenteritis (AGE), 2006-2018

## Discussion

In a generalizable sample of US ED visits from 2006 to 2018, ondansetron use for children with AGE increased markedly, with no observed decrease in intravenous rehydration or hospitalizations. Approximately half of children administered intravenous fluids or hospitalized did not receive ondansetron, suggesting that many children without dehydration receive ondansetron with limited benefit, whereas those most likely to benefit receive intravenous fluids without an adequate trial of ondansetron and ORT. Because ED visits for children with AGE are increasing, knowledge translation initiatives are urgently needed to optimize ondansetron use and reduce excessive use of intravenous fluids. Ondansetron-supported ORT for appropriately selected children can achieve intravenous rehydration rates of 9%,^6^ more than 3-fold lower than 2018 national estimates.

This study has limitations. The NHAMCS does not contain detailed patient-level information, such as dehydration severity, and longitudinal analysis of return visits is not possible. Route of medication administration is not recorded; thus, lack of benefit from ondansetron may reflect children receiving intravenous ondansetron and fluids concomitantly. Misclassification is possible; however, the NHAMCS database is rigorously quality-controlled.^4^

## Notes

Supplement. **eTable.** Inclusion and Exclusion Criteria of the Study Population, Listed by *International Statistical Classification of Diseases and Related Health Problems, Ninth Revision, Clinical Modification (ICD-9-CM)*, and *Tenth Revision (ICD-10-CM)* Codes

## References

[1] Gill PJ, Anwar MR, Thavam T, ; Pediatric Research in Inpatient Setting (PRIS) Network. Identifying conditions with high prevalence, cost, and variation in cost in US children’s hospitals. JAMA Netw Open. 2021;4(7):e2117816. doi:10.1001/jamanetworkopen.2021.17816 34309667 PMC8314139

[2] King CK, Glass R, Bresee JS, Duggan C; Centers for Disease Control and Prevention. Managing acute gastroenteritis among children: oral rehydration, maintenance, and nutritional therapy. MMWR Recomm Rep. 2003;52(RR-16):1-16. 14627948

[3] Niño-Serna LF, Acosta-Reyes J, Veroniki AA, Florez ID. Antiemetics in children with acute gastroenteritis: a meta-analysis. Pediatrics. 2020;145(4):e20193260. doi:10.1542/peds.2019-3260 32132152

[4] McCaig LF, Burt CW. Understanding and interpreting the National Hospital Ambulatory Medical Care Survey: key questions and answers. Ann Emerg Med. 2012;60(6):716-721.e1. doi:10.1016/j.annemergmed.2012.07.010 23083968

[5] Freedman SB, Hall M, Shah SS, . Impact of increasing ondansetron use on clinical outcomes in children with gastroenteritis. JAMA Pediatr. 2014;168(4):321-329. doi:10.1001/jamapediatrics.2013.4906 24566613

[6] Creedon JK, Eisenberg M, Monuteaux MC, Samnaliev M, Levy J. Reduction in resources and cost for gastroenteritis through implementation of dehydration pathway. Pediatrics. 2020;146(1):e20191553. doi:10.1542/peds.2019-1553 32487592

