# Supplementary material for: Trends in Management of Children With Acute Gastroenteritis in US Emergency Departments
Source: JAMA Netw Open. 2022 May 10;5(5):e2211201. doi: 10.1001/jamanetworkopen.2022.11201 (PMC9092198; doi:10.1001/jamanetworkopen.2022.11201)
Supplement: Supplement. — eTable. Inclusion and Exclusion Criteria of the Study Population, Listed by International Statistical Classification of Diseases and Related Health Problems, Ninth Revision, Clinical Modification (ICD-9-CM), and Tenth Revision (ICD-10-CM) Codes [file jamanetwopen-e2211201-s001.pdf]

Supplementary Online Content

Burstein B, Rogers S, Klassen TP, Freedman SB. Trends in management of children with acute gastroenteritis in US emergency departments. *JAMA Netw Open*. 2022;5(5):e2211201. doi:10.1001/jamanetworkopen.2022.11201

eTable. Inclusion and Exclusion Criteria of the Study Population, Listed by *International Statistical Classification of Diseases and Related Health Problems, Ninth Revision, Clinical Modification* (*ICD-9-CM*), and *Tenth Revision* (*ICD-10-CM*) Codes

This supplementary material has been provided by the authors to give readers additional information about their work.

© 2022 Burstein B et al. *JAMA Network Open.*

eTable. Inclusion and Exclusion Criteria of the Study Population, Listed by *International Statistical Classification of Diseases and Related Health Problems, Ninth Revision, Clinical Modification* (*ICD-9-CM*), and *Tenth Revision* (*ICD-10-CM*) Codes

Diagnostic Group	ICD-9-CM Code (2006-2015)	ICD-10-CM Code (2016-2018)
Inclusion Criteria		
Intestinal Infectious Diseases	001 Cholera<br>002 Typhoid and paratyphoid fevers<br>003 Other salmonella infections<br>004 Shigellosis<br>005 Other food poisoning<br>006 Amebiasis<br>007 Other protozoal intestinal diseases<br>008 Intestinal infections due to other organisms<br>009 Ill-defined intestinal infections	A00 Cholera<br>A01 Typhoid and paratyphoid fevers<br>A02 Other salmonella infections<br>A03 Shigellosis<br>A05 Other food poisoning<br>A06 Amebiasis<br>A07 Other protozoal intestinal diseases<br>A04 Intestinal infections due to other organisms<br>A08 Viral and other specified intestinal infections<br>A09 Ill-defined intestinal infections
Non-infectious enteritis and colitis	558.9 Other and unspecified non-infectious gastroenteritis and colitis	K52 Other and unspecified noninfective gastroenteritis and colitis
Symptoms involving digestive system	787.01 Nausea with vomiting<br>787.02 Nausea<br>787.03 Vomiting<br>787.91 Diarrhea	R11.2 Nausea with vomiting, unspecified<br>R11.0 Nausea<br>R11.1 Vomiting<br>R19.7 Diarrhea, unspecified
Gastroenteritis as a secondary diagnosis	276.51 Dehydration	E86 Dehydration
Exclusion Criteria		
Intestinal Infectious Diseases	003.2 Localized salmonella infections<br>006.3 Amebic liver abscess<br>006.4 Amebic lung abscess<br>006.5 Amebic brain abscess<br>006.6 Amebic skin ulceration	A01.0 Localized Typhoid infections<br>A02.2 Localized salmonella infections<br>A06.4 Amebic liver abscess<br>A06.5 Amebic lung abscess<br>A06.6 Amebic brain abscess<br>A06.7 Cutaneous amebiasis<br>A06.8 Amebic infection of other sites

© 2022 Burstein B et al. *JAMA Network Open.*
